# Mechanical Performance of Granite Fine Fly Dust-Filled Basalt/Glass Polyurethane Polymer Hybrid Composites

**DOI:** 10.3390/polym13183032

**Published:** 2021-09-08

**Authors:** Napisah Sapiai, Aidah Jumahat, Mohammad Jawaid, Md Zin Abu, Mochamad Chalid

**Affiliations:** 1 Faculty of Mechanical Engineering, Universiti Teknologi MARA (UiTM), Shah Alam 40450, Malaysia; napisah@uitm.edu.my; 2 Institute for Infrastructure Engineering Sustainable and Management (IIESM), Universiti Teknologi MARA, Shah Alam 40450, Malaysia; 3 Department of Biocomposite Technology, Institute of Tropical Forestry and Forest Products, Universiti Putra Malaysia Serdang, Seri Kembangan 43400, Malaysia; 4 AANS Technical & Services Sdn Bhd, Desa Manjung Raya, Lumut 32200, Malaysia; cbr1691@gmail.com; 5 Department of Metallurgical and Material Engineering, Faculty of Engineering, Kampus Baru UI, Universitas Indonesia (UI), Depok 1642, Indonesia; chalid@metal.ui.ac.id

**Keywords:** granite dust, basalt fibres, glass fibres, polymer composites, open hole tensile, low velocity impact, quasi-static indentations, interlaminar shear stress

## Abstract

The granite processing industry generates large amounts of bottom granite dust waste every day. After the drying and heating process of concrete mixture production, the granite dust is blown and collected in the filtering nozzle. This very fine particle granite dry fly dust, with a particle size maximum distribution of 500 μm, can easily be blown away by wind and cause serious environmental impacts. The use of this waste material would be an effective way to reduce such impacts. Therefore, this paper presents an experimental study on the potential of granite dust as a filler in enhancing the mechanical performance of a hybrid basalt/glass (WB/GCSM) composite. The unhole and open hole tensile (UHT and OHT) properties, low velocity impact (LVI) properties, quasi-static indentations (QSI) properties, flexural properties, interlaminar shear stress (ILSS) properties, and morphology of the developed WB/GCSM composites were evaluated. To meet the objective of this study, composite specimens were produced using 1.5–60 μm granite fly dust at three (3) different loadings (1, 3 and 5 wt%). This granite fly dust was incorporated into polyurethane resin using a mechanical stirring technique. The production of FRP laminates then completed using a hand lay-up and vacuum bagging technique. Four types of the WB/GCSM composites systems, i.e., [WB/GCSM], [WB/GCSM/1GD], [WB/GCSM/3GD] and [WB/GCSM/5GD] were fabricated and compared. The analysis results for the mechanical tests revealed that the incorporation of granite dust of up to 3 wt% had increased the UHT, OHT, LVI, QSI, flexural and ILSS properties of all WB/GCSM composites systems. Higher levels of damage tolerance in UHT and OHT tests, and increased ductility index in the LVI test were obtained when granite dust was added up to 5 wt%. However, a remarkable improvement in all mechanical properties was noticed for [WB/GCSM/1GD], which recorded the highest mechanical performance among all WB/GCSM composite systems.

## 1. Introduction

Basalt fibres have gained great attention as a reinforcing material in polymer composite industries because they are chemically stable, with excellent mechanical and thermal properties. Basalt fibres are made from basalt rocks, which consist of SiO_2_, Al_2_O_3_, CaO, MgO, Fe_2_O_3_, and FeO as the main components [1,2,3,4,5]. Thus, these mineral-based natural fibres are non-toxic, eco-friendly, easy to recycle, and inexpensive. The main components of basalt fibres are similar to glass fibres, but with superior mechanical strength, thermal stability, and chemical resistance, which make them a great alternative for glass fibres. However, it is well-known that composite materials are vulnerable to failure due to the inherent brittleness of both the fibres and the matrix.

In general, hybrid composites are advanced engineered materials, consisting of two or more materials that are embedded or reinforced within a matrix. Hybrid composites are the key in developing innovative solutions by mixing two or more materials to achieve a synergistic effect. Subsequently, superior properties within the hybrid material can be obtained, such as improved elastic modulus, strength, ductility, and lighter weight [6]. These hybridisation qualities are widely reported by previous researchers [6,7,8,9,10]. For example, Bulut and Erklig [6] concluded that the hybridisation of two or three different fibres can significantly affect indentation responses, i.e., force and absorbed energy. Sapiai et al. [7,8] and Muhamad et al. [9] found that the hybridisation of kenaf fibre with glass fibre can produce better composite properties compared to the single kenaf fibre system. Al-Hajaj et al. [11] and Dhakal et al. [12] have proven that the hybridisation of carbon fibres with flax can significantly improve the environmental, thermal, and mechanical performance of the composite. In addition to hybridisation with different fibres, the incorporation of fillers within a matrix have been demonstrated as being able to enhance the elastic modulus, strength, and toughness of composites, without sacrificing the strain to failure and thermal stability. Thermoset polymers used as a matrix in composites have always exhibited poor crack resistance, brittle fractures, and crystalline structures, which ultimately reduce their mechanical performance. Many researchers have overcome this problem by adding fillers/nanofillers. The use of fillers to modify the matrix is an alternative method to improve mechanical, thermal, and dynamic properties without altering the weight or processability of composites [13]. Several types of fillers can be used to modify composites, such as metal oxides (alumina, iron oxide, magnesium hydroxide, and titanium dioxide) [13,14,15], nanomaterials (nanosilica, nanoclay, graphene, and carbon nanotubes) [7,16,17,18], rubber [19,20], and thermoplastic [21,22].

Granite dust is a waste material that can potentially be used as a reinforcement due to its excellent properties, such as high modulus of elasticity and strength [23,24,25,26]. Granite dust is also classified as an industrial waste that can threaten the environment. A large amount of granite waste can form colloidal waste in water when granite stones undergo processing by the granite processing industry [27]. The use of granite dust is relevant in the present time, especially for the development of innovative technology, which would overcome problems associated with its disposal, including environmental problems. Granite dust is primarily composed of alumina, silica, and potassium, with small amounts of magnesium and calcium. Owing to its chemical composition (i.e., alumina, silica, and magnesium, which are excellent fillers), granite dust has the potential of being used in polymer composites. Awad et al. [26] investigated the effect of different granite dust weight percentages on the flexural properties of HDPE composites. It was indicated that 50 wt% of granite dust in HDPE can increase flexural strength, while a weight percentage of higher than 50 wt% can lead to particle agglomeration, which would reduce the performance of the composites. Subhash et al. [28] claimed that 40 wt% of granite dust in epoxy composites can be unsuitable for fabrication due to improper wetting between granite particles and epoxy resin at higher concentration. The results indicated that the Vickers hardness was increased with up to 20 wt% of granite dust, while impact strength was increased with up to 30 wt% of granite dust. The percentage of moisture content was also increased with increasing granite dust content due to the porosity of the composites.

A review of the previous literature showed that no specific work has been conducted to investigate the modifying effect of granite dust in the polyurethane matrix on the mechanical properties of basalt/glass composites. Most granite dust research was developed in various construction applications and building materials using bottom fine granite aggregate to replace natural sand and cement in concrete, filler material for roads, and manufacturing bricks and tiles for construction, infrastructure, and building [23,24,25,26,27]. Most of this research used bottom granite dust that is collected after the grinding or cutting process of granite stone; meanwhile, in this research, the granite dust used is a fine fly dry granite dust that is collected from the filter after drying, blowing, and heating at an elevated temperature of 200 °C during the preparation of concrete mixture at the quarry plant. Therefore, in this research, the fine fly granite dust will be used as a filler in enhancing the mechanical behaviour of basalt/glass composites to achieve the comprehensive application of granite dust. The granite dust-filled basalt/glass composites were designed in a unique arrangement for damaged or cracked surfaces and pipeline repair as a patch to replace carbon fibre reinforced composite patch. The effects of the various mechanical properties, i.e., (UHT, OHT, LVI, QSI, flexural, ILSS properties) were studied by different loading of granite dust, while the form and types of the fibre were specified for woven-type basalt fibre and chopped strand mat glass fibre. The addition of granite dust came at no cost because it is a waste material from the granite processing industry and can be considered as utilisation of waste material to solve disposal problems. This research also aims to further develop the potential of using granite dust in natural material-based composites.

## 2. Experimental Procedure

### 2.1. Materials

**Figure 1:**
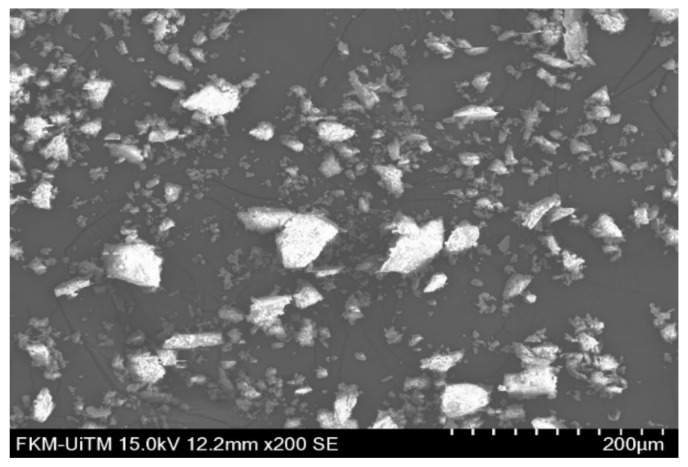
SEM image of granite dust.

Hybrid basalt/glass (WB/GCSM) composites were developed using different loadings of micron sized granite waste powder, a twill weave (TW) type of basalt fibres, a chopped strand mat (CSM) type of glass fibre and polyurethane resin as the matrix. Polyurethane (Konudur 250 OM-PL Sommerharz) was used in this study, which is a low viscosity organic-mineral resin supplied by MC-Bauchemie, Bottrop, Germany with a ratio of 2:1 (resin: hardener). Twill weave basalt fibres were supplied by Zhejiang GBF Basalt Fibre Co. Ltd., Dongyang, China, while the chopped strand mat glass fibres were supplied by Vistec Technology, Puchong, Malaysia. Granite dust was collected from Jabatan Kerja Raya (JKR), Kelantan Branch, Malaysia. Figure 1 shows the morphology of granite dust under a Scanning Electron Microscope (SEM, Hitachi, Tokyo, Japan). The SEM image indicated that the granite particles have irregular shapes, with diameters ranging between 1.5–60 µm. Table 1 lists the chemical composition of granite dust obtained using an X-ray fluorescence (XRF, Bruker, Billerica, MA, USA) spectrometer, which is mainly composed of silica (SiO_2_) and alumina (Al_2_O_3_).

### 2.2. Fabrication of Composites

**Figure 2:**
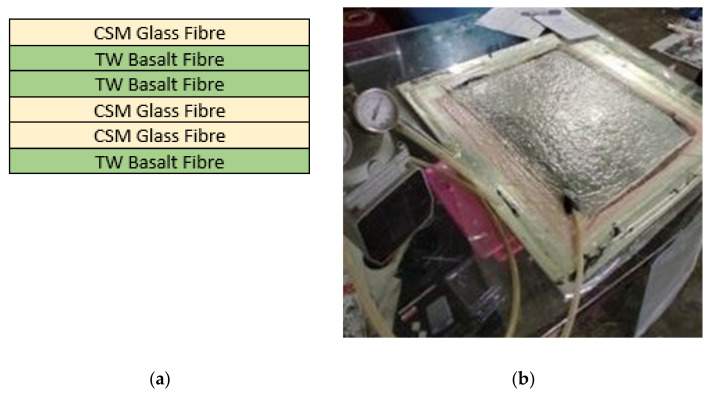
Fabrication setup of WB/GCSM composites: (**a**) fibre sheets arrangement; and (**b**) vacuum bagging system.

To fabricate the WB/GCSM composites, a polyurethane mixture was prepared with different weight percentages of granite dust at 1, 3 and 5 wt% using a mechanical stirrer. The glass and basalt fibres were cut into 300 × 300 mm^2^ sheets and layered up with polyurethane/granite dust-filled polyurethane resin on top of each other to form a laminate composite. Rolling was employed to eliminate trapped air. Each laminate composite comprised three plies of CSM glass fibre and three plies of TW basalt fibre, as shown in Figure 2a. The granite dust-filled basalt/glass composites comprised three plies of CSM glass fibre and three plies of basalt fibre, whereby 4mm of thickness can be obtained from the fibre ply arrangement. All laminate composites are prepared under vacuum bagging, as shown in Figure 2b. This study has synthesised four types of WB/GCSM composites, coded as [WB/GCSM], [WB/GCSM/1GD], [WB/GCSM/3GD] and [WB/GCSM/5GD] based on different weight percentages of the added granite dust.

### 2.3. Mechanical Tests

#### 2.3.1. Unhole Tensile (UHT) and Open Hole Tensile (OHT) Test

The tensile test was conducted as per the Standard Test Method for Open-Hole Tensile Strength of Polymer Matrix Composite Laminates (ASTM D5766). The standard specimen size for tensile test should be followed ASTM D3039/D3039M—08. However, for the OHT, some requirement needs to be concerned and followed as per standard ASTM D 5766. The thickness of the specimen in this study is chosen according to a hole diameter to thickness ratio (D/h) of 1.5. The hole with 6 mm was drilled in the middle of the specimen. Therefore, the ratio (D/h) = (6/4) = 1.5. The WB/GCSM composites, size of 300 × 36 × 4 mm^3^, were tested using an Instron 3382 Universal Testing Machine with a crosshead speed of 2 mm/min. A hole measuring 6 mm in diameter was drilled into the composites to determine the damage tolerance properties of the specimens under tensile loading.

#### 2.3.2. Low Velocity Impact (LVI) Test

The impact test was performed according to Standard Test Method for Measuring the Damage Resistance of a Fiber-Reinforced Polymer Matrix Composite to a Drop-Weight Impact (ASTM D7136). The composite specimens were cut into 50 × 50 × 4 mm^3^ strips using a vertical bending machine. An Instron Dynatup 8250 Drop Weight Impact Tester, with a 13 mm in diameter hemispherical tip impactor, was used to determine the load carrying capabilities, energy absorbed, deflection, ductility index and impact strength of the composite specimens.

#### 2.3.3. Quasi-Static Indentation (QSI) Test

The static indentation resistance properties evaluated by Standard Test Method for Measuring the Damage Resistance of a Fiber-Reinforced Polymer-Matrix Composite to a Concentrated Quasi-Static Indentation Force (ASTM D6264). The WB/GCSM composites were cut into 50 × 50 × 4 mm^3^ strips and tested using an Instron 3382 Universal Testing Machine. A 13 mm indenter was applied at 2 mm/min cross head speed during testing. A constant force was applied until the indenter had fully penetrated the specimens. The total energy absorption was calculated based on the area under the graph of force versus displacement.

#### 2.3.4. Flexural Test

The composite specimens with sizes of 80 × 13 × 4 mm^3^ were tested using an Instron 3382 Universal Testing Machine with a crosshead speed of 2 mm/min according to Standard Test Methods for Flexural Properties of Unreinforced and Reinforced Plastics and Electrical Insulating Materials (ASTM D790). The span length to sample thickness ratio was maintained at 16:1. Approximately five specimens for each WB/GCSM composites systems were tested and the average flexural strength and modulus were calculated from the obtained values.

#### 2.3.5. Interlaminar Shear Strength (ILSS) Test

The interlaminar shear strength test was conducted using an Instron 3382 Universal Testing Machine according to Standard Test Method for Short-Beam Strength of Polymer Matrix Composite Materials and Their Laminates (ASTM D2344). All the composite specimens were cut into 36 × 12 × 4 mm^3^ strips. This test was conducted at a crosshead speed of 2 mm/min and approximately five specimens for each composite laminate were tested to obtain an average value of ILSS.

## 3. Results and Discussion

### 3.1. Unhole Tensile (UHT) and Open Hole Tensile (OHT) Properties

**Figure 3:**
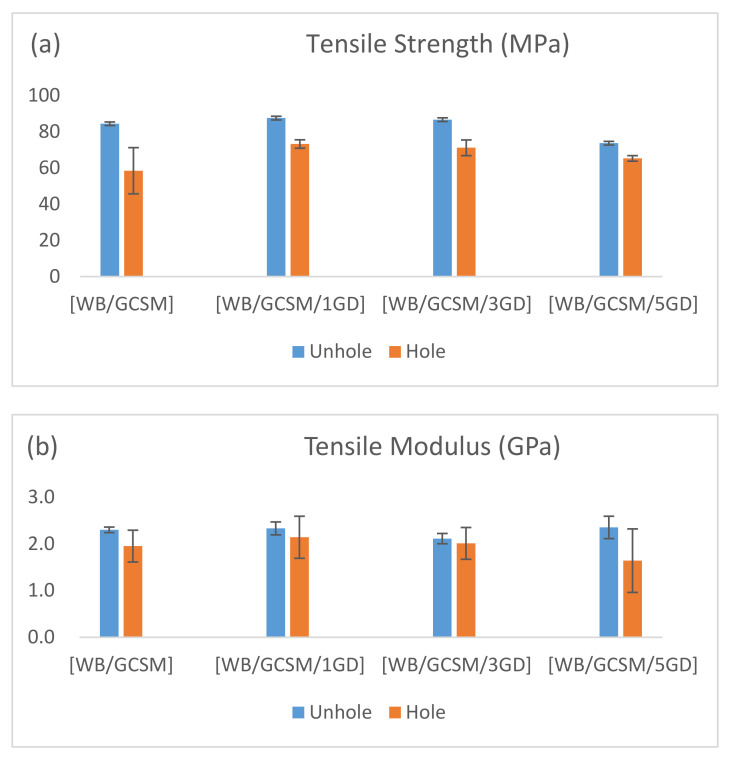
UHT and OHT properties of [WB/GCSM], [WB/GCSM/1GD], [WB/GCSM/3GD] and [WB/GCSM/5GD] composites (**a**) Tensile strength and (**b**) Tensile modulus.

The variations of the UHT and OHT properties of WB/GCSM composites are characterised as a function of granite dust content, as depicted in Figure 3. The values of the tensile properties are summarised in Table 2. A slight improvement can be seen with 1.0 wt% of granite dust loading [WB/GCSM/1GD], which showed an increase of 3.72% for UHT strength and 1.30% for UTH modulus compared to without granite dust loading [WB/GCSM]. At 3.0 wt% of granite dust loading [WB/GCSM/3GD], the UTH strength of the specimen was increased by 2.68%, yet the UTH modulus was deceased by 8.60% compared to the values for [WB/GCSM]. When granite dust loading was increased to 5.0 wt% [WB/GCSM/5GD], the decreasing trend was observed for both strength and modulus values. These results showed that the optimum interaction between basalt/glass fibres and granite dust-filled epoxy resin occurred at the lowest granite dust loading of 1.0 wt%. The [WB/GCSM/1GD] composite was the strongest material, in terms of strength and modulus, among all WB/GCSM composite systems. The increasing filler loading has decreased the modulus and strength of the composites by propagating the formation of ductile materials. Similar trends were reported by numerous researchers when they added more fillers/nanofillers to modify the polymer matrix. The reduction in mechanical performance would occur because of the agglomeration tendency of the fillers within the matrix, acting as stress concentration points and inducing crack formation. The mechanical performance of the composites can be affected by several parameters, such as the dispersion and distribution of fillers, the compatibility between filler and matrix, and the interfacial bonding between filler and matrix [7,13,14,15,16].

As for the OHT properties of the WB/GCSM composites, different percentages of granite dust loading have led to different tensile properties. The results showed that OHT strength was increased with increasing granite dust loading by 25.27% for [WB/GCSM/1GD], 21.72% for [WB/GCSM/3GD] and 11.66% for [WB/GCSM/5GD] compared to the OHT strength of [WB/GCSM]. When 1.0 and 3.0 wt% of granite dust loadings were added, the OHT modulus was increased by 9.74% and 3.08% for [WB/GCSM/1GD] and [WB/GCSM/3GD], respectively. However, when granite dust loading was increased to 5.0 wt%, the OHT modulus of [WB/GCSM/5GD] was decreased by 15.90% compared to the OHT modulus of [WB/GCSM]. This uncertain trend in UHT and OHT properties might be due to structural flaws that occurred during specimen fabrication.

**Figure 4:**
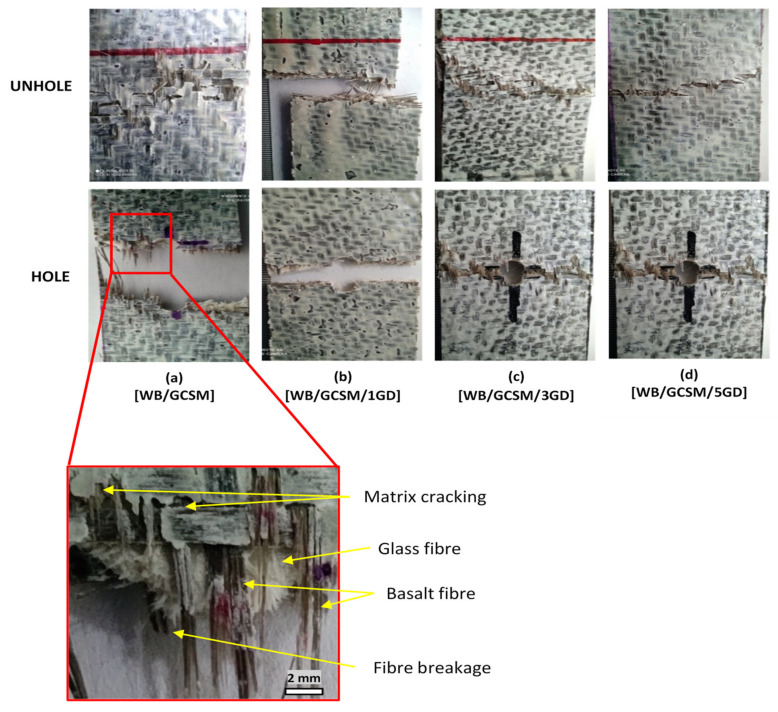
UHT and OHT fracture analysis of [WB/GCSM], [WB/GCSM/1GD], [WB/GCSM/3GD] and [WB/GCSM/5GD] composites after being subjected to tensile test.

As shown in Figure 4, most of the UHT composite specimens break near the grip/end tab, which is at the lowest strength of the composite itself, while the OHT composite specimens break at the hole area, where the hole has created localised stress in the composite specimens. This portrayed that the appearance of a hole can lead to a higher stress concentration in the surrounding area, which breaks the specimen. Although an uncertain trend was observed, the results have also shown that [WB/GCSM/1GD], with 1.0 wt% of granite dust loading, has the best tensile properties among WB/GCSM composites, which indicated that granite dust can enhance the tensile performance of the composite specimens. The significant ability of granite dust in modifying resin was also proven by the increasing damage tolerance and decreasing stress reduction indexes when more granite dusts were embedded in WB/GCSM composites. The damage tolerance indexes for [WB/GCSM], [WB/GCSM/1GD], [WB/GCSM/3GD], and [WB/GCSM/5GD] were 69.27%, 83.65%, 82.11%, and 88.60%, respectively. Meanwhile, the stress reduction indexes were recorded at 30.73% for [WB/GCSM], 16.35% for [WB/GCSM/1GD], 17.895% for [WB/GCSM/3GD], and 11.40% for [WB/GCSM/5GD].

Figure 4 shows the failure mechanism of the UHT and OHT properties of WB/GCSM composites after the tensile test. It was observed that the WB/GCSM composites experienced brittle fracture behaviour, whereby the delamination mode failure occurring due to fibre breakage and matrix cracking.

### 3.2. Low Velocity Impact (LVI) Properties

**Figure 5:**
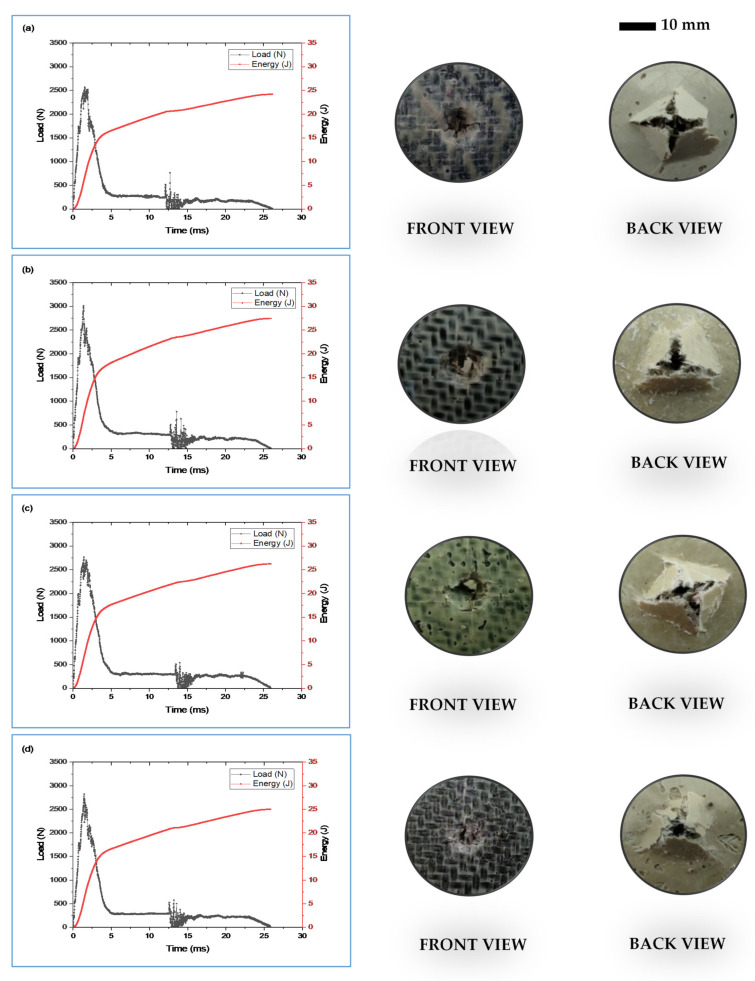
Typical force–time and energy–time curves: (**a**) [WB/GCSM]; (**b**) [WB/GCSM/1GD]; (**c**) [WB/GCSM/3GD]; and (**d**) [WB/GCSM/1GD], with corresponding images of the front and back view after the impact test.

The impact properties of the WB/GCSM composites can be characterised in terms of the maximum load and energy absorbed, supported by damage evaluation after being subjected to an impact test. In other words, impact properties were determined when the composite specimens have absorbed and dissipated the strain energy through various failure modes, such as matrix cracking, fibre breakage and delamination. Figure 5 shows the load-time and energy–time curves, with corresponding damage images of the WB/GCSM composites. The load–time curves of WB/GCSM composites, with and without granite dust, showed similar behaviours, initially with a linear behaviour and sharp peak found between 1.80 and 2.30 ms. The sharp drop in the load implied that a small amount of initiate energy was absorbed. However, all composite specimens have absorbed more energy during the damage propagation process. Oscillation peaks were also observed for all composite samples, indicating the propagation of cracks before the composite specimens completely failed. The [WB/GCSM/1GD] composite recorded the highest peak load, followed by [WB/GCSM/5GD], [WB/GCSM/3GD], and [WB/GCSM] composites. The highest peak is the maximum force that the material can withstand under the specific impact energy [3]. The [WB/GCSM/1GD] composite has also shown a higher total energy absorbed at 23.33 J, which was an increase of 14.53% compared to the energy absorbed by [WB/GCSM]. The total energy absorbed by [WB/GCSM/3GD] and [WB/GCSM/5GD] was also increased by 9.67% and 2.90%, respectively. The inclusion of granite dust has also contributed towards increasing the impact performance of the WB/GCSM composites. The significance of granite dust addition was also interpreted using the ductility index (DI). The ductility index is an indication of the brittle behaviour of the composite material [29]. Thus, a low DI value would indicate that the composite material has become more brittle. In this study, WB/GCSM composites incorporated with granite dust have higher DI values compared to [WB/GCSM]. Consequently, the [WB/GCSM] composite was more brittle than [WB/GCSM/1GD], [WB/GCSM/3GD], and [WB/GCSM/5GD]. The brittleness of the composites would lead to a reduction in impact energy, as lesser load energy would lead to failure and low ductility. Therefore, it was concluded that the inclusion of granite dust will reduce brittleness (referring to ductility index), increase stiffness (as indicated in the elastic region), and increase energy absorbed (as calculated from the area under the graph). All these effects are exaggerated to reduce impact damage through the resistance to damage progression. The impact performance of the WB/GCSM composites is summarised in Table 3.

In general, all WB/GCSM composite specimens experienced similar failure mechanisms, which occurred as localised indentation on the front face and a bulge deformation at the back face. Damage images have revealed matrix cracking, delamination of fibres, fibre breakage, and fibre pull-out. As reported by Hajaj et al. [11], fibre breakage and pull-out could occur at the front face of the impacted specimen due to high stress and sudden indentation. Meanwhile, matrix cracks at the back face of the impacted specimen could occur due to impact and bending stress. The matrix cracking initiation and propagation would subsequently induce delamination at the fibre interfaces, and the delamination growth depends on the interface strength between adjacent plies.

### 3.3. Quasi-Static Indentation (QSI) Properties

**Figure 6:**
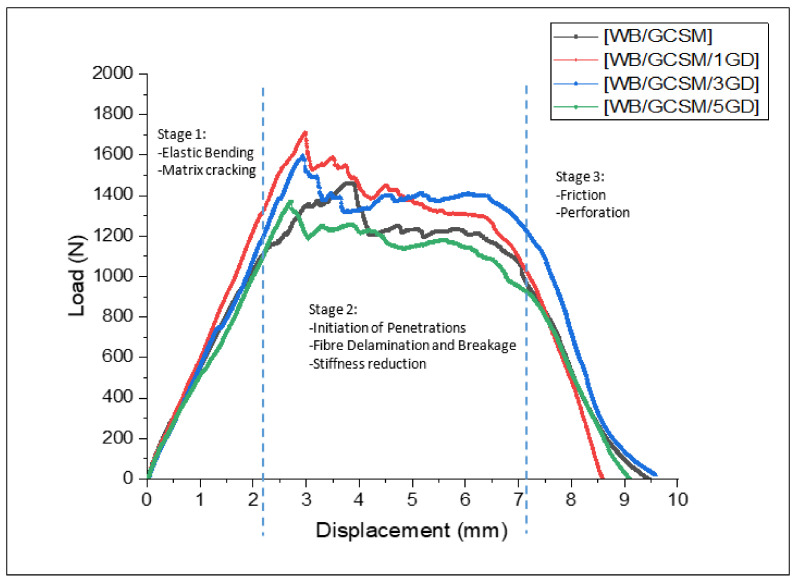
Typical load–displacement of [WB/GCSM], [WB/GCSM/1GD], [WB/GCSM/3GD] and [WB/GCSM/5GD] composites.

**Figure 7:**
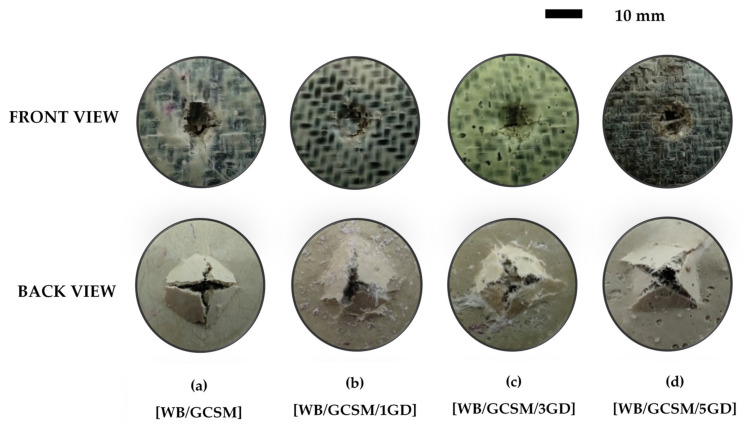
Damage fracture of [WB/GCSM], [WB/GCSM/1GD], [WB/GCSM/3GD] and [WB/GCSM/5GD] composites after being subjected to QSI test.

Figure 6 shows the load–displacement curves of WB/GCSM composites, which represent the QSI behaviour of the composites during tests. It is clear that all the curves showed similar trends, in terms of indentation responses, which can be divided into three stages. The first stage refers to the elastic bending stage, whereby the matrix would start to crack with increasing load. The second stage is known as the damage stage, whereby the composites would reach the highest load (maximum load), which would reduce stiffness and start to delaminate. The point of the first drop of force is the damage initiation point, regardless of whether maximum strength has been achieved or not. After the first force drop point, with the following displacement increase, the fluctuations of force were observed due to fibre breakage, which occurred in every ply of composite. It was determined that the load carrying capacity of the composites was decreased. This phenomenon was also reported and discussed by Bulut and Erklig [6,30]. In the last stage, the composite laminates have completely been perforated and penetrated. For QSI behaviour, the energy absorbed was calculated from the area under the load–displacement curves. The relationship between maximum load and energy absorbed by WB/GCSM composites when granite dust is added as shown in Table 4. The results showed that the maximum load for [WB/GCSM/1GD] and [WB/GCSM/3GD] was increased by 19.86% and 8.22%, respectively, while the energy absorbed by these specimens has increased by 5.36% for [WB/GCSM/1GD] and 9.56% for [WB/GCSM/3GD] compared to [WB/GCSM]. However, the QSI performance was reduced with 5 wt% of granite dust within the WB/GCSM composites. The damage behaviours of the composites when subjected to LVI and QSI loading were relatively similar to each other as can be seen in Figure 5 and Figure 7, respectively. Damage behaviours observed for WB/GCSM composites, such as matrix cracking, debonding, fibre delamination, and breakage were anticipated based on peak frequency ranges. These types of damage mechanism were also reported elsewhere [6,31,32].

### 3.4. Flexural Properties

**Figure 8:**
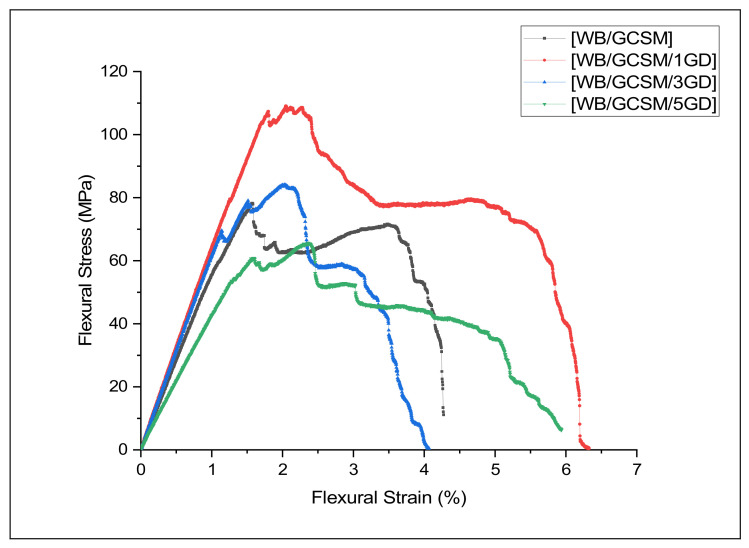
Typical flexural stress–strain curves for [WB/GCSM], [WB/GCSM/1GD], [WB/GCSM/3GD], and [WB/GCSM/5GD] composites.

The typical flexural stress–strain responses of the WB/GCSM composites are illustrated in Figure 8. The flexural modulus, flexural strength and flexural strain of the composites, as derived from the stress–strain curves, were depicted and summarised in Table 5. Figure 8 and Table 5 show that the flexural strength and modulus of WB/GCSM composites has significantly improved, with the addition of 1 and 3 wt% of granite dust. The [WB/GCSM/1GD] composite, which, with the addition of 1 wt%, showed the highest flexural properties among the WB/GCSM composite systems. The [WB/GCSM/1GD] composite achieved 109.07 MPa for flexural strength and 6.47 GPa for flexural modulus, which were increments of 39.42% and 12.33%, respectively, compared to the [WB/GCSM] composite. As for the [WB/GCSM/3GD] composite, the added 3 wt% of granite dust loading has also improved the flexural strength and modulus by 6.26% and 8.33%, respectively, compared to WB/GCSM composites. However, the flexural strength and modulus of this specimen were reduced by 23.79% and 3.55%, respectively, compared to the [WB/GCSM/1GD] composite. Meanwhile, the flexural strength and modulus of the [WB/GCSM/5GD] composite were shown to be the lowest among the hybrid [WB/GCSM] composite systems, which indicated that the higher amount of granite dust has reduced the flexural performance of the composites.

### 3.5. Interlaminar Shear Strength (ILSS) Properties

**Figure 9:**
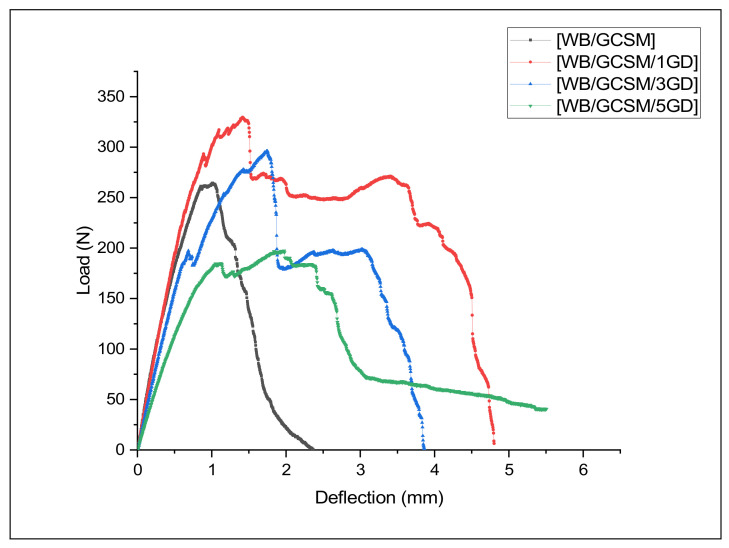
Typical ILSS load-deflection curves for [WB/GCSM], [WB/GCSM/1GD], [WB/GCSM/3GD] and [WB/GCSM/5GD] composites.

**Figure 10:**
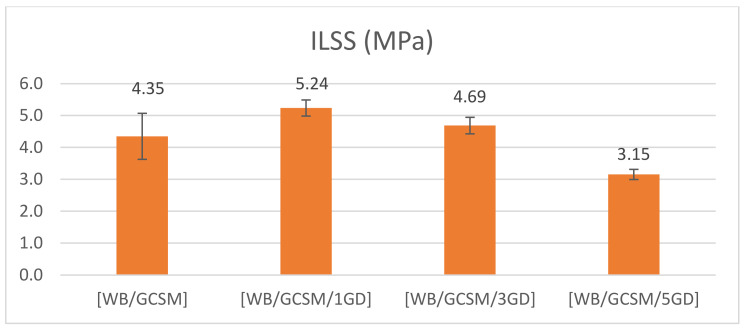
Interlaminar shear strength of [WB/GCSM], [WB/GCSM/1GD], [WB/GCSM/3GD] and [WB/GCSM/5GD] composites.

Figure 9 shows the typical load–defection curves of [WB/GCSM], [WB/GCSM/1GD], [WB/GCSM/3GD], and [WB/GCSM/5GD] composites, as obtained from the ILSS test. These curves have similar patterns as the flexural properties and QSI behaviour, whereby linear behaviour was initiated (elastic region) and fluctuated load was carried to the middle until the composite completely failed. As previously discussed in the QSI section, the fluctuating load can be used to determine the response of the composites when load is applied, whereby at the early stage, the matrix started to crack, followed by fibre delamination and fibre breakage/rupture. The [WB/GCSM/1GD] composite indicated the highest maximum load, followed by [WB/GCSM/3GD], [WB/GCSM], and [WB/GCSM/5GD] composites. Figure 10 shows that the ILSS is increased by 20.46% for the [WB/GCSM/1GD] composite and 7.82% for the [WB/GCSM/3GD] composite compared to the [WB/GCSM] composite. It was concluded that the addition of 1.0 and 3.0 wt% of granite dust has improved the ILSS of the WB/GCSM composites. As similarly found in other properties (UHT and OHT, flexural, and QSI), the addition of 5 wt% of granite dust has also reduced the ILSS. The reduced mechanical performance when nanofillers modify composite materials has been discussed by numerous researchers [13,16,26,28]. A similar reason was reported, whereby the agglomerated structure of the modified matrix resin caused stress concentration, thus leading to composite failure. Furthermore, the higher filler loadings could also affect the dispersibility of fillers within the matrix resin during the fabrication process due to a higher filler loading can increase the viscosity of modified resin. Hence, this will lead to improper bonding and decrease wettability resulting in poor interface adhesion between the fibres, filler, and matrix resin. In ILSS testing, various mechanisms such as tension, compression, and shearing take place simultaneously. Higher filler loading decreases the resistance to shear due to poor interfacial adhesion, and thus leads to composite deficiency.

## 4. Conclusions

This study has strived to develop and enhance the mechanical performance of WB/GCSM composites via modification with a waste material, i.e., granite dust. In this study, four systems of WB/GCSM composites, namely, [WB/GCSM], [WB/GCSM/1GD], [WB/GCSM/3GD], and [WB/GCSM/5GD] were successfully fabricated and characterised. All WB/GCSM composite systems showed similar behaviours, with and without the addition of granite dust, based on the load–displacement curves and the damage fractures after being tested. Based on the flexural, QSI, and ILSS results, the load–displacement of the WB/GCSM composites started with the elastic behaviour (linear curve). Then, the matrix started to crack with the increase of load, followed by fibre delamination and ruptures (fluctuation curve) until the composite completely failed. In conclusion, the [WB/GCSM/1GD] composite has the highest mechanical performance compared to the other WB/GCSM composites. The addition of 1.0 wt% of granite dust [WB/GCSM/1GD] has increased the UHT strength by 3.72%, UHT modulus by 1.30%, LVI energy absorbed by 14.53%, QSI energy absorbed by 5.36%, flexural strength by 39.42%, flexural modulus by 12.33%, and ILSS by 20.46% compared to the specimen without the granite dust [WB/GCSM] composite. The addition of 3 wt% of granite dust [WB/GCSM/3GD] composite has also enhanced the mechanical performance, but not as well as the [WB/GCSM/1GD] composite. The higher addition of granite dust of up to 5 wt% has reduced the mechanical performance of the [WB/GCSM/5GD] composites. The addition of granite dust within WB/GCSM composites has also reduced the stress reduction index, increased the damage tolerance index and ductility index, which confirmed the granite dust’s significant contribution. The utilisation of granite dust may have the potential to embark into other fields as a reinforcing material.

## Figures and Tables

**Table 1:** Chemical composition of granite dust obtained via XRF analysis.

Composition	SiO_2_	Al_2_O_3_	K_2_O	CaCo_3_	Fe_2_O_3_	MnO	TiO_2_	SO_3_
Percentage (%)	78.91	10.52	6.07	2.07	1.73	0.18	0.10	0.08

**Table 2:** Summarised UHT and OHT properties of [WB/GCSM], [WB/GCSM/1GD], [WB/GCSM/3GD] and [WB/GCSM/5GD] composites.

Composites	Tensile Properties							
	Tensile Strength, *σ_t_* (MPa)		Tensile Modulus, *E_t_* (GPa)		Tensile Strain at Break, *ε_f_* (%)		Damage Tolerance σ_hole_/σ_unhole_ (%)	Strength Reduction (100 − σ_hole_/σ_unhole_) (%)
	Unhole	Hole	Unhole	Hole	Unhole	Hole		
[WB/GCSM]	84.40 ± 5.92	58.46 ± 12.74	2.30 ± 0.06	1.95 ± 0.34	3.99 ± 0.36	3.19 ± 0.02	69.27	30.73
[WB/GCSM/1GD]	87.54 ± 6.35	73.23 ± 2.33	2.33 ± 0.14	2.14 ± 0.45	3.51 ± 0.37	3.19 ± 0.24	83.65	16.35
[WB/GCSM/3GD]	86.66 ± 10.93	71.16 ± 4.33	2.11 ± 0.11	2.01 ± 0.34	3.20 ± 0.02	2.98 ± 0.17	82.11	17.89
[WB/GCSM/5GD]	73.68 ± 22.58	65.28 ± 1.51	2.04 ± 0.24	1.64 ± 0.68	3.00 ± 0.47	3.56 ± 0.45	88.60	11.40

**Table 3:** Impact properties of [WB/GCSM], [WB/GCSM/1GD], [WB/GCSM/3GD] and [WB/GCSM/5GD] composites.

Composites	Impact Properties					
	Peak Load (N)	Deflection at Peak Load (mm)	Total Energy Absorbed, E_t_ (J)	Initiation Energy, E_m_ (J)	Propagation Energy, E_p_ (J)	Ductility Index, E_p_/E_m_
[WB/GCSM]	2623.7 ± 221.73	5.41 ± 0.18	20.37 ± 1.26	8.63 ± 0.67	11.74	1.36
[WB/GCSM/1GD]	3010.7 ± 249.44	4.29 ± 0.18	23.33 ± 1.43	6.75 ± 0.12	16.58	2.46
[WB/GCSM/3GD]	2763.8 ± 144.20	4.12 ± 0.71	22.34 ± 1.06	6.46 ± 1.06	15.88	2.46
[WB/GCSM/5GD]	2820.6 ± 350.90	4.21 ± 0.65	20.96 ± 4.83	5.98 ± 0.62	14.98	2.51

**Table 4:** Quasi-static indentation (QSI) properties of [WB/GCSM], [WB/GCSM/1GD], [WB/GCSM/3GD], and [WB/GCSM/5GD] composites.

Composites	Quasi-static Indentation Properties		
	Maximum Load (kN)	Displacement (mm)	Energy Absorbed (J)
[WB/GCSM]	1.46 ± 0.29	3.84 ± 0.60	8.58 ± 0.31
[WB/GCSM/1GD]	1.71 ± 0.06	2.99 ± 0.22	9.04 ± 0.08
[WB/GCSM/3GD]	1.58 ± 0.03	3.64 ± 0.67	9.40 ± 0.02
[WB/GCSM/5GD]	1.37 ± 0.07	2.71 ± 0.57	8.38 ± 0.50

**Table 5:** Flexural properties of [WB/GCSM], [WB/GCSM/1GD], [WB/GCSM/3GD], and [WB/GCSM/5GD] composites.

Composites	Flexural Properties		
	Flexural Modulus (GPa)	Flexural Strength (MPa)	Flexural Strain at Break (%)
[WB/GCSM]	5.76 ± 0.62	78.23 ± 10.95	1.57 ± 1.52
[WB/GCSM/1GD]	6.47 ± 0.51	109.07 ± 8.72	2.04 ± 0.01
[WB/GCSM/3GD]	6.24 ± 0.43	83.13 ± 11.43	2.15 ± 0.20
[WB/GCSM/5GD]	4.28 ± 0.45	65.66 ± 2.36	2.36 ± 0.41

## Data Availability

The data presented in this study are available upon request from the corresponding author.

## Abbreviations

WB/GCSM	Woven Basalt/Glass Chopped Stand Mat
UHT	Unhole Tensile
OHT	Open Hole Tensile
LVI	Low Velocity Impact
QSI	Quasi-Static Indentations
ILSS	Interlaminar Shear Stress
GD	Granite Dust
TW	Twill Weave
CSM	Chopped Stand Mat
SEM	Scanning Electron Microscope
XRF	X-ray fluorescence
ASTM	American Society for Testing and Materials
JKR	Jabatan Kerja Raya
